# Acupuncture-based interventions for blood pressure control and cardiovascular risk-related outcomes in adults with hypertension: a systematic review and meta-analysis of randomized controlled trials

**DOI:** 10.3389/fmed.2026.1888399

**Published:** 2026-08-12

**Authors:** Xiangwei Zhong, Chengdong Yu, Haoyan Guo

**Affiliations:** 1 Affiliated Guangdong Integrated Traditional Chinese and Western Medicine Hospital, Guangzhou University of Chinese Medicine, Foshan, China; 2 Guangzhou University of Chinese Medicine, Guangzhou, China

**Keywords:** Acupoint stimulation, acupuncture, blood pressure, electroacupuncture, hypertension, systematic review

## Abstract

Blood pressure control remains difficult in many adults with hypertension, and safe non-pharmacological strategies that can support conventional care are clinically relevant. This systematic review and meta-analysis evaluated acupuncture-based interventions for blood pressure control and cardiovascular risk-related outcomes in adults with hypertension. Randomized studies were identified from major biomedical databases and trial registers from inception to 16 March 2026. The primary synthesis focused on penetrating acupuncture and electroacupuncture, while non-penetrating acupoint stimulation was examined in extended analyses. Risk of bias was assessed using RoB 2, and certainty of evidence was evaluated using GRADE. Sixteen study reports representing 15 independent randomized studies were included. For end-of-treatment office blood pressure, acupuncture-based interventions reduced systolic blood pressure (MD −6.63 mmHg, 95% CI: −11.41 to −1.84) but showed no clear effect on diastolic blood pressure (MD −0.07 mmHg, 95% CI: −9.54 to 9.39). Change-score analyses showed a significant reduction in office diastolic blood pressure (MD −3.97 mmHg, 95% CI: −7.26 to −0.68), while systolic change crossed the null. Ambulatory blood pressure evidence was sparse; 24-h ambulatory diastolic change favored acupuncture-based interventions. No confirmed acupuncture- or stimulation-related serious adverse event was clearly reported. Overall, acupuncture-based interventions may support blood pressure control, especially systolic office blood pressure and diastolic change outcomes.

## 1 Introduction

Hypertension remains one of the most important modifiable risk factors for cardiovascular disease worldwide. Large-scale global analyses show that elevated blood pressure contributes substantially to cardiovascular morbidity and mortality, while hypertension awareness, treatment, and control remain suboptimal in many populations (1, 2). Contemporary guidelines emphasize accurate blood pressure measurement, lifestyle modification, and pharmacological treatment when indicated, because blood pressure lowering reduces major cardiovascular events across a wide range of baseline risk profiles (3–6). However, long-term blood pressure management is often challenged by adherence, treatment tolerability, patient preference, residual lifestyle risk, and the need for sustained self-management. These challenges have increased interest in safe, acceptable, and integrable non-pharmacological strategies that may support conventional hypertension care.

Acupuncture-based interventions have been investigated as potential adjunctive or supportive approaches for blood pressure control. These interventions include manual acupuncture, electroacupuncture, auricular or abdominal acupuncture, and non-penetrating acupoint stimulation such as transcutaneous electrical acupoint stimulation. Their proposed physiological effects include modulation of autonomic balance, reduction of sympathetic outflow, central autonomic regulation, and improvement of stress-related or sleep-related pathways that may influence blood pressure regulation (7, 8). Recent lifestyle and hypertension guidance increasingly recognizes the importance of patient-centered non-pharmacological management, but acupuncture-based interventions remain difficult to interpret clinically because the intervention modality, stimulation intensity, comparator design, and treatment exposure differ substantially across trials (9).

Previous systematic reviews and meta-analyses have suggested that acupuncture may reduce blood pressure in patients with hypertension, but the certainty and clinical applicability of this evidence remain debated (10–12). Several more recent reviews have examined primary hypertension, older adults, ambulatory blood pressure, or broader non-pharmacological interventions, generally reporting favorable but heterogeneous findings (13–16). A key challenge is that different evidence streams are often pooled together, including penetrating acupuncture, electroacupuncture, acupoint stimulation, office blood pressure, ambulatory blood pressure, final values, change scores, sham-controlled trials, and usual-care-controlled trials. Such heterogeneity limits the direct usefulness of pooled estimates for cardiovascular decision-making.

This systematic review and meta-analysis therefore aimed to evaluate the effects of acupuncture-based interventions on blood pressure control and cardiovascular risk-related outcomes in adults with hypertension. The primary synthesis focused on penetrating acupuncture and electroacupuncture, while non-penetrating acupoint stimulation was examined in extended analyses. We separately assessed office blood pressure, 24-h ambulatory blood pressure, final values, change scores, safety outcomes, and certainty of evidence. By distinguishing intervention type and blood pressure outcome type, this review sought to provide a more clinically interpretable summary of the randomized evidence relevant to hypertension management.

## 2 Materials and methods

### 2.1 Design and reporting Standards

This systematic review and meta-analysis was conducted and reported in accordance with the Preferred Reporting Items for Systematic Reviews and Meta-Analyses 2020 statement and the PRISMA literature search extension (17, 18). The review question, eligibility criteria, outcome hierarchy, data extraction items, risk-of-bias assessment, certainty-of-evidence assessment, and statistical procedures were specified before study screening and data extraction. Acupuncture intervention characteristics were extracted according to the revised Standards for Reporting Interventions in Clinical Trials of Acupuncture (19). Risk of bias in randomized studies was assessed using the revised Cochrane risk-of-bias tool for randomized trials, and the certainty of evidence was evaluated using the Grading of Recommendations Assessment, Development and Evaluation approach (20, 21).

### 2.2 Eligibility criteria

#### 2.2.1 Study design

Eligible studies were randomized controlled studies published as full-text peer-reviewed journal articles. Parallel-group randomized studies with individual participant allocation were eligible. Multiple reports from the same randomized study were linked under a single study label and counted once in quantitative synthesis. Quasi-randomized studies, non-randomized clinical studies, observational studies, before–after studies without a randomized control group, case reports, case series, protocols, conference abstracts, dissertations, animal studies, narrative reviews, systematic reviews, and meta-analyses were excluded.

#### 2.2.2 Participants

Eligible participants were adults aged 18 years or older with primary hypertension, essential hypertension, untreated hypertension, mild hypertension, or study-defined hypertension. Studies enrolling patients with secondary hypertension, gestational hypertension, pulmonary hypertension, hypertensive emergency, acute stroke, acute coronary syndrome, postoperative blood pressure elevation, or dialysis-related blood pressure instability were excluded. Studies enrolling mixed elevated-blood-pressure populations, including prehypertension or stage I hypertension, were retained only for prespecified sensitivity analysis when hypertension-only data were not separately extractable. Studies enrolling broader populations with blood pressure outcomes but unclear hypertension-only eligibility were retained only for broad sensitivity analysis and did not contribute to the primary hypertension-focused synthesis.

#### 2.2.3 Interventions

Eligible interventions were acupuncture-based blood pressure interventions delivered at named acupuncture points or acupoint regions. Penetrating acupuncture interventions included manual body acupuncture, electroacupuncture, abdominal acupuncture, auricular acupuncture involving needle insertion or acupuncture-like stimulation, and other needle-based acupoint protocols. Non-penetrating electrical acupoint stimulation, including transcutaneous electrical acupoint stimulation, was classified as acupoint stimulation and analyzed separately from penetrating acupuncture.

The primary intervention synthesis was restricted to penetrating acupuncture and electroacupuncture studies. Non-penetrating acupoint stimulation studies contributed to the extended acupoint-stimulation analysis and sensitivity analyses. Studies evaluating acupressure alone, auricular seed pressing alone, laser acupuncture alone, moxibustion alone, cupping alone, tuina alone, herbal medicine, qigong, yoga, meditation, or exercise as the only active intervention were excluded. Studies in which acupuncture was combined with another active non-pharmacological therapy that was not delivered equally to the control group were excluded from the primary synthesis.

#### 2.2.4 Comparators

Eligible comparators were sham acupuncture, non-acupoint needling, sham acupoint stimulation, waiting-list control, no-treatment control, usual care, lifestyle advice, antihypertensive drug therapy, and conventional antihypertensive management without active acupuncture or active acupoint stimulation. Studies comparing two active acupuncture prescriptions without a non-acupuncture, sham, usual-care, or routine-treatment control were excluded from pairwise meta-analysis.

#### 2.2.5 Outcomes

The primary outcomes were end-of-treatment office systolic blood pressure and end-of-treatment office diastolic blood pressure, measured in mmHg. The primary effect measure was the between-group mean difference in end-of-treatment office blood pressure. Secondary blood pressure outcomes were change from baseline in office systolic blood pressure, change from baseline in office diastolic blood pressure, final 24-h ambulatory systolic blood pressure, final 24-h ambulatory diastolic blood pressure, change from baseline in 24-h ambulatory systolic blood pressure, change from baseline in 24-h ambulatory diastolic blood pressure, and study-defined blood pressure control or treatment response. Cardiovascular risk-related and patient-centered outcomes were heart rate variability, sleep quality, anxiety symptoms, and health-related quality of life. Safety outcomes were total adverse events, serious adverse events, acupuncture- or stimulation-related adverse events, treatment discontinuation, and withdrawal due to adverse events. The primary time point was the final measurement at completion of the planned intervention course. Short-protocol or single-session studies were retained and analyzed in short-term sensitivity analyses.

### 2.3 Information sources and search strategy

A comprehensive literature search was conducted in PubMed/MEDLINE, Embase, Cochrane Central Register of Controlled Trials, Web of Science Core Collection, Scopus, CINAHL, AMED, ClinicalTrials.gov, and the World Health Organization International Clinical Trials Registry Platform from database inception to 16 March 2026. The search strategy combined terms for hypertension, acupuncture-based interventions, acupoint stimulation, and randomized controlled studies.

The hypertension search block included “hypertension,” “essential hypertension,” “primary hypertension,” “high blood pressure,” “mild hypertension,” and “blood pressure.” The intervention search block included “acupuncture,” “manual acupuncture,” “electroacupuncture,” “auricular acupuncture,” “abdominal acupuncture,” “scalp acupuncture,” “acupoint,” “acupoint stimulation,” and “transcutaneous electrical acupoint stimulation.” The trial design search block included “randomized controlled trial,” “randomised controlled trial,” “random allocation,” “randomized,” “randomised,” “sham,” and “placebo.” Reference lists of included articles and relevant prior reviews were searched to identify additional randomized studies. Trial registry records were used to identify linked publications and duplicate reports; registry records without full-text peer-reviewed journal publication were excluded.

### 2.4 Study selection

All retrieved records were imported into a reference-management database and duplicates were removed. Two reviewers independently screened titles and abstracts, assessed full-text articles, and determined eligibility according to the predefined criteria. Each record was coded as excluded or retained at the title-and-abstract stage and at the full-text stage. Disagreements were resolved by a third reviewer. Full-text exclusion reasons were recorded and summarized in the PRISMA flow diagram.

### 2.5 Data extraction

Two reviewers independently extracted data using a standardized extraction form. Extracted bibliographic and design information included first author, publication year, country or region, study setting, trial design, number of randomized participants, number of analyzed or completed participants, and trial registration status. Participant-level information included eligibility population, hypertension type or severity, baseline medication status, and the sample used for each extractable blood pressure analysis. Intervention information included acupuncture or acupoint-stimulation modality, acupoint prescription, needle insertion or stimulation method, electroacupuncture or TEAS parameters, retention or stimulation time, treatment frequency, treatment duration, total number of sessions, and relevant co-interventions. Comparator information included sham procedure, non-acupoint procedure, usual care, waiting-list control, antihypertensive medication, and treatment exposure in the control arm. Outcome data included means, standard deviations, sample sizes, event counts, total analyzed participants, and measurement time points. Blood pressure values reported in units other than mmHg were converted to mmHg. For studies reporting standard errors, confidence intervals, or exact *p* values, standard deviations were calculated algebraically. For multi-arm studies with more than one eligible acupuncture-based intervention arm and one shared control arm, each eligible intervention arm was treated as a separate comparison and the shared control group was divided evenly across comparisons. For multiple reports from the same randomized study, blood pressure outcomes were extracted from the main clinical report, and secondary patient-centered outcomes were extracted from companion reports without duplicating participant counts.

### 2.6 Risk of Bias assessment

Risk of bias was assessed independently by two reviewers using RoB 2 (21). The assessment covered bias arising from the randomization process, bias due to deviations from intended interventions, bias due to missing outcome data, bias in measurement of the outcome, and bias in selection of the reported result. Each domain was judged as low risk of bias, some concerns, or high risk of bias. The overall risk of bias for each outcome was determined according to the highest level of concern across domains. Disagreements were resolved by a third reviewer. Risk-of-bias judgments were presented using summary and traffic-light plots.

### 2.7 Certainty of evidence

The certainty of evidence was assessed using the GRADE approach (20). Randomized controlled evidence started as high-certainty evidence. The certainty of evidence was downgraded for risk of bias, inconsistency, indirectness, imprecision, and publication bias. The final certainty of evidence for each outcome was classified as high, moderate, low, or very low.

GRADE assessment was conducted for end-of-treatment office systolic blood pressure, end-of-treatment office diastolic blood pressure, change in office systolic blood pressure, change in office diastolic blood pressure, change in 24-h ambulatory systolic blood pressure, change in 24-h ambulatory diastolic blood pressure, blood pressure control or treatment response, heart rate variability, health-related quality of life, and safety and tolerability. The results were summarized in a GRADE Summary of Findings table.

### 2.8 Data synthesis and statistical analysis

The principal comparison was acupuncture-based intervention versus sham procedure, no-treatment control, waiting-list control, usual care, control acupoints, or conventional antihypertensive management without active acupuncture or active acupoint stimulation. The primary synthesis was restricted to penetrating acupuncture and electroacupuncture studies. TEAS and other non-penetrating acupoint stimulation studies were analyzed in the extended acupoint-stimulation analysis and in sensitivity analyses.

Office blood pressure final values, office blood pressure change scores, 24-h ambulatory blood pressure final values, and 24-h ambulatory blood pressure change scores were synthesized separately. Continuous blood pressure outcomes measured in mmHg were pooled as mean differences with 95% confidence intervals. Continuous outcomes measured using different instruments were planned to be pooled as standardized mean differences with 95% confidence intervals, but heterogeneous measurement and sparse reporting led to narrative synthesis for heart rate variability, quality of life, sleep, and anxiety outcomes. Dichotomous outcomes were pooled as risk ratios with 95% confidence intervals when definitions and reporting were sufficiently comparable; otherwise, they were summarized narratively.

Random-effects models were used for the primary meta-analyses because clinical and methodological heterogeneity was expected across acupuncture protocols, comparator designs, baseline medication status, hypertension severity, and outcome measurement methods. Between-study variance was estimated using restricted maximum likelihood, and confidence intervals for pooled effects were calculated using the Hartung–Knapp adjustment (22, 23). Heterogeneity was quantified using τ^2^, Cochran’s *Q*, and the *I*^2^ statistic (24). Single-study estimates were shown descriptively without heterogeneity metrics. Statistical significance was defined as a two-sided *p* < 0.05.

### 2.9 Prespecified subgroup and sensitivity analyses

Prespecified subgroup and sensitivity analyses examined intervention type, comparator type, baseline medication status, treatment duration, total number of treatment sessions, short-protocol studies, TEAS inclusion, elevated-blood-pressure populations, broad non-hypertension-only populations, and statistical model choice. Intervention type was categorized as manual acupuncture, electroacupuncture, auricular or abdominal acupuncture, and non-penetrating acupoint stimulation. Comparator type was categorized as sham or sham-acupoint control, waiting-list or no-treatment control, usual care, and antihypertensive medication control. Baseline medication status was categorized as medication-free or untreated, stable antihypertensive medication, and mixed or unclear medication status. Treatment duration was categorized as less than 8 weeks or 8 weeks and longer. Total number of sessions was categorized as fewer than 20 sessions or 20 sessions and more.

Sensitivity analyses repeated the main blood pressure analyses after excluding short-protocol studies, including TEAS studies, including elevated-blood-pressure sensitivity studies, including broad non-hypertension-only sensitivity studies, and using fixed-effect models.

### 2.10 Assessment of small-study effects and publication Bias

All pooled outcome sets contained fewer than 10 independent comparisons. Therefore, funnel plot asymmetry tests were not conducted. The decision not to perform formal small-study-effect testing followed standard recommendations that regression-based asymmetry tests require a sufficient number of studies for interpretable results (25, 26). Publication bias was incorporated qualitatively into the GRADE assessment for each key outcome.

### 2.11 Software

All statistical analyses were performed using R with the meta and metafor packages (23, 27). Forest plots, risk-of-bias plots, subgroup analyses, extended acupoint-stimulation analyses, and sensitivity analyses were generated from the same analytic dataset.

## 3 Results

### 3.1 Study selection and evidence base

**Figure 1:**
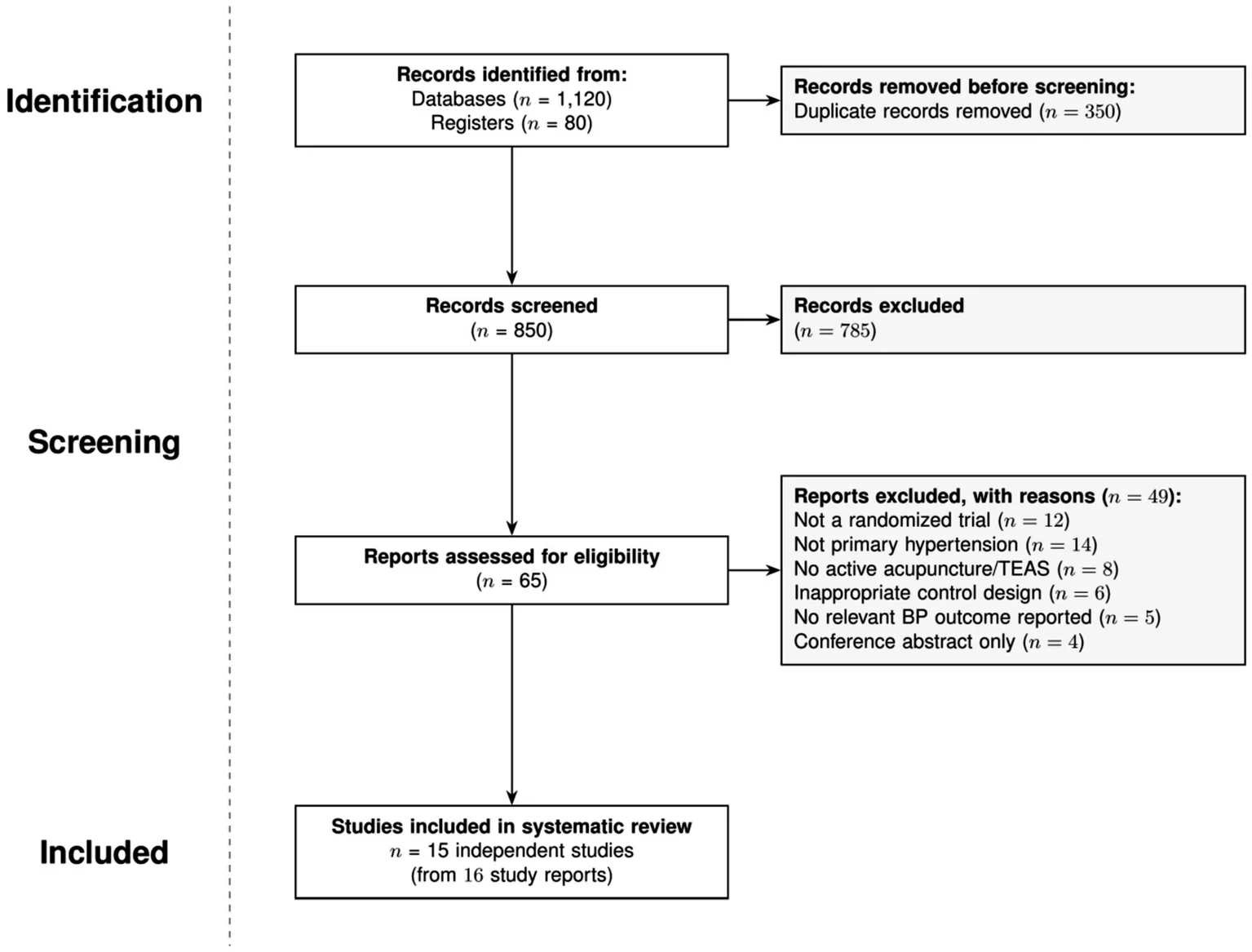
PRISMA study selection flow diagram. The flowchart summarizes the identification, screening, eligibility assessment, and inclusion process. Sixteen study reports representing 15 independent randomized studies were included in the systematic review.

The database and register searches identified 1,200 records, including 1,120 records from databases and 80 records from registers. After removal of 350 duplicate records, 850 records were screened by title and abstract, of which 785 were excluded. Sixty-five reports were assessed for eligibility, and 49 were excluded. Finally, 16 study reports representing 15 independent randomized studies were included in the systematic review (Figure 1).

The included reports were organized according to their contribution to the evidence synthesis (Table 1). The primary synthesis focused on penetrating acupuncture or electroacupuncture studies with extractable office or ambulatory blood pressure outcomes. TEAS studies were retained for the extended acupoint-stimulation analysis. Huang et al. (40, 41) were treated as companion reports of the same randomized study, with the 2022 report contributing only secondary patient-centered outcomes.

**Table 1:** Included study reports and data contribution.

Study report	Intervention category	Main data contribution	Use in synthesis
Macklin et al. (32)	Penetrating acupuncture	Office BP change and safety	Primary change-score synthesis
Flachskampf et al. (33)	Penetrating acupuncture	24-h ambulatory BP, office BP, safety	Ambulatory BP synthesis
Yin et al. (34)	Penetrating acupuncture	Office final BP, office BP change, response, safety	Primary office BP synthesis
Kim et al. (35)	Penetrating acupuncture	Ambulatory BP and office BP	Ambulatory BP synthesis
Liu et al. (36)	Penetrating acupuncture; elevated-BP sensitivity	Office BP change, HRV, quality of life, safety	Elevated-BP sensitivity only
Li et al. (37)	Electroacupuncture	24-h average BP change, longitudinal BP, mechanistic outcomes	Ambulatory BP change synthesis
Abdi et al. (38)	Abdominal or auricular acupuncture; broad sensitivity	Broad sensitivity; not hypertension-only primary	Broad sensitivity only
Zheng et al. (39)	Penetrating acupuncture	Ambulatory BP change, BP variability, safety	Ambulatory BP synthesis
Huang et al. (40)	Penetrating acupuncture	Office BP, HRV, safety	Primary office BP synthesis
Huang et al. (41)	Companion report	QOL, ADL, pain, patient-centered outcomes	Secondary outcomes only; not counted as an additional study
Maheshkumar et al. (42)	Penetrating acupuncture; short-term	Short-term office BP response	Primary change-score synthesis; short-term sensitivity
Zhang et al. (43)	Penetrating acupuncture; short-term	End-of-treatment/short-protocol office BP and mechanism	Primary final-office synthesis; short-term sensitivity
Tu et al. (44)	TEAS	Extended acupoint-stimulation analysis	Extended analysis only
Ma et al. (45)	TEAS	Extended acupoint-stimulation analysis	Extended analysis only
Bao et al. (46)	TEAS	Extended acupoint-stimulation analysis	Extended analysis only
Emara et al. (47)	TEAS	Extended acupoint-stimulation analysis	Extended analysis only

ADL, activities of daily living; BP, blood pressure; CI, confidence interval; HRV, heart rate variability; QOL, quality of life; TEAS, transcutaneous electrical acupoint stimulation. Huang et al. (40, 41) were companion reports of the same randomized study; Huang et al. (41) was used only for secondary patient-centered outcomes and was not counted as an additional independent study.

### 3.2 Characteristics of included studies and participants

The characteristics of the included studies and participants are summarized in Table 2. The 15 independent randomized studies included 1,846 randomized participants, with sample sizes ranging from 29 to 428 participants. The studies were conducted in the United States, Germany, the Republic of Korea, Iran, China, India, and Egypt. Most studies enrolled adults with primary, essential, mild, or untreated hypertension, while a small number of studies enrolled broader elevated-blood-pressure or overweight/obesity populations with blood pressure outcomes and were therefore retained only for sensitivity or extended analyses. Medication status varied across studies. Some studies enrolled medication-free or untreated participants, whereas others included participants receiving stable antihypertensive medication, usual care, or routine medical therapy. Comparator arms included sham acupuncture, sham acupoint procedures, control acupoints, waiting-list or usual-care controls, and routine antihypertensive treatment alone. This clinical and methodological diversity supported the use of separate primary, ambulatory, extended-intervention, and sensitivity analyses.

**Table 2:** Characteristics of included studies and participants.

Study	Country/ region	Population and sample used	Medication status	Control/comparator arm
Macklin et al. (32)	United States	Untreated hypertension; 192 randomized; 188 analyzed for the primary BP outcome	Medication-free	Sham acupuncture
Flachskampf et al. (33)	Germany	Mild-to-moderate hypertension; 160 randomized; 140 completed treatment	Mixed or stable antihypertensive medication	Sham acupuncture
Yin et al. (34)	Republic of Korea	Essential hypertension or elevated BP; 41 randomized; 30 completed	Stable antihypertensive medication	Sham acupuncture
Kim et al. (35)	Republic of Korea	Essential hypertension; 33 randomized; 28 completed	Not consistently reported	Sham acupuncture
Liu et al. (36)	Republic of Korea	Prehypertension or stage I hypertension; 30 randomized and analyzed	Medication-free or untreated	No-treatment/control procedure
Li et al. (37)	United States	Hypertension; 65 randomized and analyzed	Medication-free	Control acupoints
Abdi et al. (38)	Iran	Overweight/obesity population with BP outcomes; hypertension-only eligibility unclear; 400 randomized; 330 completed	Mixed or unclear	Control/sham condition
Zheng et al. (39)	China, English report	Mild hypertension; 428 randomized; 415 ITT; active-vs-sham contrast sample extracted according to outcome	Medication-free mild hypertension	Sham acupuncture or waiting-list
Huang et al. (40, 41)	Taiwan, China	Older adults with hypertension in home health care; 70 randomized; 62 completed	Stable antihypertensive drugs	Antihypertensive drugs alone
Maheshkumar et al. (42)	India	Hypertension on medication; 90 randomized; 80 included in BP table	Stable antihypertensive drugs	Sham control
Zhang et al. (43)	China, English report	Essential hypertension; 29 randomized and analyzed	Not consistently reported	LR3 plus sham acupoint
Tu et al. (44)	China, English report	Hypertension; 60 randomized and analyzed	Usual care or antihypertensive drugs	Usual care
Ma et al. (45)	China, English report	Mild hypertension; 120 randomized and analyzed	Not consistently reported	Usual care/control condition
Bao et al. (46)	China, English report	Hypertension with anxiety or sleep disorder; 88 randomized; analyzable sample extracted by outcome	Routine treatment	Routine treatment
Emara et al. (47)	Egypt	Primary hypertension; 40 randomized and analyzed	Routine medical therapy	Routine therapy alone

BP, blood pressure; ITT, intention-to-treat. Huang et al. (40, 41) were companion reports of the same randomized study and were summarized as Huang et al. (40, 41).

### 3.3 Characteristics of acupuncture-based interventions

The characteristics of acupuncture-based interventions are summarized in Table 3. Eleven studies evaluated needle-based acupuncture interventions, including manual acupuncture, electroacupuncture, abdominal electroacupuncture, auricular acupoint intervention, and short-protocol single- or paired-acupoint stimulation. Four studies evaluated non-penetrating TEAS protocols and were retained for the extended acupoint-stimulation analysis. Commonly used acupoints or acupoint regions included LR3, LI4, LI11, ST36, PC6, SP6, KI3, ST40, and abdominal or auricular acupoint regions. Treatment exposure varied substantially across studies. Most multi-session acupuncture protocols delivered treatment over 4 to 12 weeks, with frequencies ranging from once weekly to five sessions per week. Short-term acupuncture protocols included single-session or short-protocol designs and were handled separately in sensitivity analyses. TEAS studies generally used repeated stimulation sessions over 4 to 12 weeks. This heterogeneity in modality, acupoint selection, session frequency, and total treatment exposure supported separate interpretation of primary acupuncture analyses and extended acupoint-stimulation analyses.

**Table 3:** Characteristics of acupuncture-based interventions.

Study	Modality	Main acupoints or regions	Treatment exposure
Macklin et al. (32)	Manual acupuncture	Individualized, standardized, or control prescriptions	30-min sessions, twice weekly, over 6–8 weeks; up to 12 sessions
Flachskampf et al. (33)	Manual acupuncture	Chinese-medicine antihypertensive prescription	Needles retained for 20 min; visits lasted about 30 min; 5 sessions/week for 2 weeks, then 3 sessions/week for 4 weeks; 22 sessions in total
Yin et al. (34)	Manual acupuncture	TCM antihypertensive acupoint prescription	Approximately twice weekly over 8 weeks; about 16 sessions; retention time not consistently extractable
Kim et al. (35)	Manual acupuncture	Bilateral ST36 and PC6	20-min sessions, twice weekly, over 8 weeks; 16 sessions in total
Liu et al. (36)	Manual acupuncture	LI11, SP4, ST36, LR3, PC6	20-min sessions, twice weekly, over 8 weeks; 16 sessions in total
Li et al. (37)	Electroacupuncture	PC5–PC6 plus ST36–ST37; control acupoints included LI6–LI7 plus GB37–GB39	30-min sessions, once weekly, over 8 weeks; 8 sessions in total
Abdi et al. (38)	Abdominal electroacupuncture or auricular acupoint intervention	Abdominal and auricular acupoint regions	Twice weekly over 6 weeks; 12 sessions in total; retention or stimulation time not consistently extractable
Zheng et al. (39)	Manual acupuncture	Affected-meridian acupuncture protocol	30-min sessions, three times weekly, over 6 weeks; 18 sessions in total
Huang et al. (40, 41)	Manual acupuncture	Bilateral SP10, SP6, LR3, ST36, LI4	30-min sessions, twice weekly, over 12 weeks; 24 sessions in total
Maheshkumar et al. (42)	Manual acupuncture	LR3	Single 20-min session
Zhang et al. (43)	Manual acupuncture	LR3 and KI3	Short-protocol acupuncture session; detailed treatment exposure was handled as short-term sensitivity evidence
Tu et al. (44)	TEAS	Bilateral LI4 and LI11 home-based stimulation	Home-based stimulation, four sessions weekly, over 12 weeks; 48 planned sessions
Ma et al. (45)	TEAS	Frequency-specific acupoint stimulation protocol	30-min sessions, three times weekly, over 4 weeks; 12 sessions in total
Bao et al. (46)	TEAS	LR3, LI4, LI11, ST36, PC6, PC4	30-min sessions, three times weekly, over 4 weeks; 12 sessions in total
Emara et al. (47)	TEAS	Acupoint stimulation protocol	40-min sessions, three times weekly, over 6 weeks; 18 sessions in total

TCM, traditional Chinese medicine; TEAS, transcutaneous electrical acupoint stimulation. Comparator information is reported in Table 2. Abdi et al. (38) was retained for broad sensitivity analysis because the intervention combined abdominal electroacupuncture and auricular acupoint intervention in an overweight/obesity-related population with blood pressure outcomes.

### 3.4 Risk of Bias assessment

**Figure 2:**
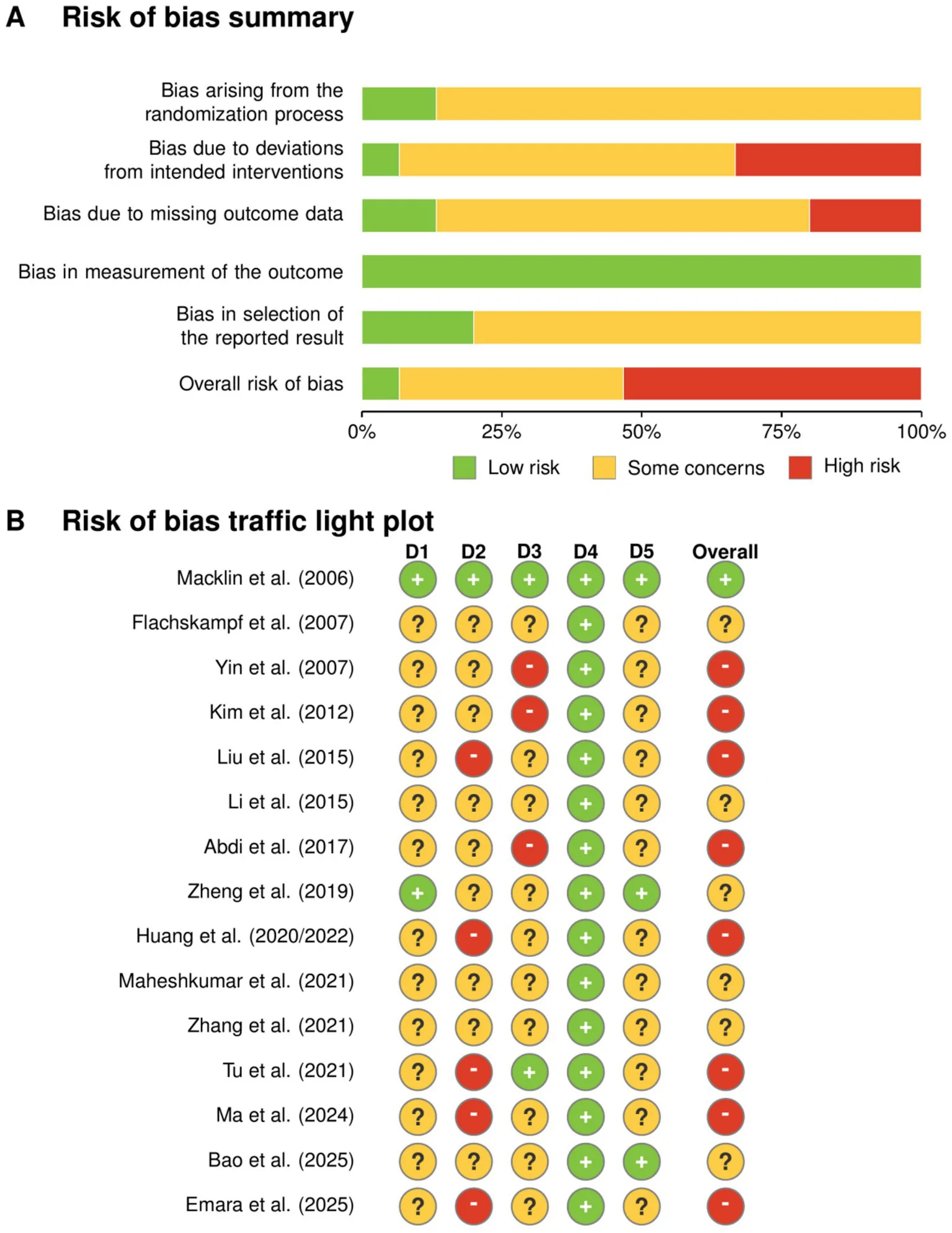
Risk-of-bias assessment of the included randomized studies. **(A)** Risk-of-bias summary showing review authors’ judgments for each RoB 2 domain as percentages across 15 independent studies. **(B)** Study-level risk-of-bias traffic light plot. D1, Bias arising from the randomization process; D2, Bias due to deviations from intended interventions; D3, Bias due to missing outcome data; D4, Bias in measurement of the outcome; D5, Bias in selection of the reported result. Symbols indicate low risk (+), some concerns (?), and high risk (−).

The risk-of-bias assessment is summarized in Figure 2. Among the 15 independent studies, one study was judged as low risk of bias overall, six studies were judged as having some concerns, and eight studies were judged as high risk of bias. The most frequent concerns were related to deviations from intended interventions, missing outcome data, and selection of the reported result. Bias in measurement of the outcome was judged as low risk across all studies because blood pressure was measured as an objective clinical outcome. In contrast, subjective outcomes such as quality of life, pain, sleep, and anxiety were more vulnerable to lack of blinding and incomplete reporting. Overall, the risk-of-bias profile supported downgrading the certainty of evidence for the main blood pressure and patient-centered outcomes.

### 3.5 Effects on office blood pressure and cardiovascular risk-related outcomes

**Figure 3:**
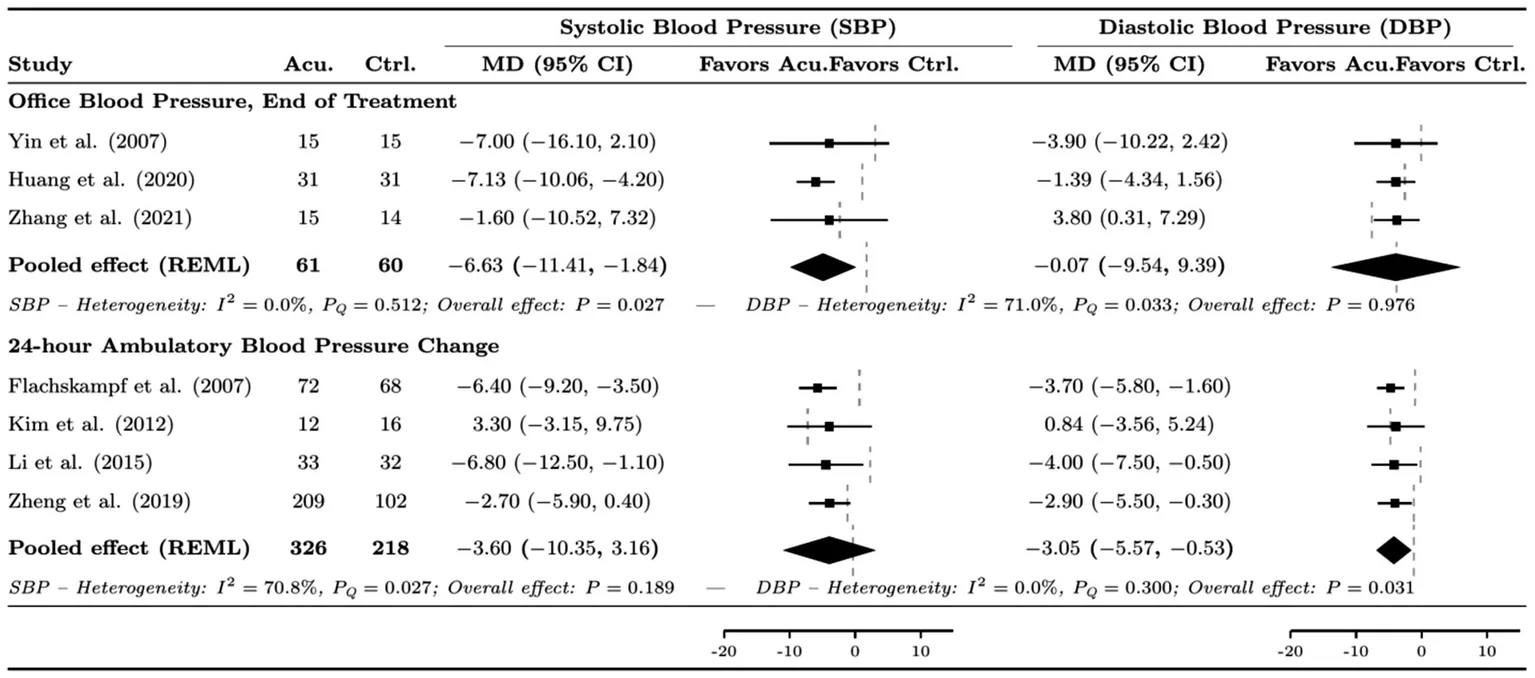
Forest plots of the effects of acupuncture-based interventions on office and ambulatory blood pressure outcomes. SBP, systolic blood pressure; DBP, diastolic blood pressure; MD, mean difference; CI, confidence interval; REML, restricted maximum likelihood. Acu. and Ctrl. denote the sample sizes in the acupuncture-based intervention and control groups, respectively. Pooled estimates were calculated using random-effects models with the Hartung–Knapp adjustment. Negative MD values favor acupuncture-based interventions. The upper panel presents office blood pressure at the end of treatment, and the lower panel presents change from baseline in 24-h ambulatory blood pressure. Squares represent individual study estimates, diamonds represent pooled effects, and horizontal lines indicate 95% CIs. The vertical dashed line at zero indicates no between-group difference.

The pooled effects on blood pressure and cardiovascular risk-related outcomes are summarized in Figure 3 and Table 4. For end-of-treatment office blood pressure, three studies with 121 participants contributed to the primary final-value synthesis. Acupuncture-based interventions were associated with lower office SBP compared with control conditions (MD −6.63 mmHg, 95% CI: −11.41 to −1.84; *p* = 0.027), with no observed heterogeneity (*I*^2^ = 0.0%). In contrast, the corresponding estimate for office DBP showed no clear between-group difference (MD −0.07 mmHg, 95% CI: −9.54 to 9.39; *p* = 0.976), with substantial heterogeneity (*I*^2^ = 71.0%). For office blood pressure change from baseline, four studies with 360 participants were included. The pooled estimate for office SBP change favored acupuncture-based interventions but crossed the null (MD −7.78 mmHg, 95% CI: −16.47 to 0.91; *p* = 0.065), with high heterogeneity (*I*^2^ = 88.8%). The pooled estimate for office DBP change favored acupuncture-based interventions (MD −3.97 mmHg, 95% CI: −7.26 to −0.68; *p* = 0.031), although heterogeneity remained moderate to substantial (*I*^2^ = 69.7%). Ambulatory blood pressure evidence was more limited. Two studies with 168 participants contributed to the final-value 24-h ambulatory blood pressure analysis, and the pooled estimates for both SBP and DBP were highly imprecise. For change from baseline in 24-h ambulatory blood pressure, four studies with 544 participants were included. The pooled estimate for ambulatory SBP change favored acupuncture-based interventions but crossed the null (MD −3.60 mmHg, 95% CI: −10.35 to 3.16; *p* = 0.189), whereas ambulatory DBP change showed a significant reduction (MD −3.05 mmHg, 95% CI: −5.57 to −0.53; *p* = 0.031), with no observed heterogeneity (I^2^ = 0.0%). Blood pressure control or response, heart rate variability, health-related quality of life, sleep, and anxiety outcomes were not pooled because definitions, measurement instruments, intervention types, and reporting formats varied across studies. These outcomes were therefore summarized narratively and interpreted as supportive cardiovascular risk-related evidence rather than primary efficacy endpoints.

**Table 4:** Summary of pooled effects on blood pressure and cardiovascular risk-related outcomes.

Outcome	Studies	Participants	Effect measure	Pooled effect	I^2^	*p* value
End-of-treatment office SBP	3	121	MD, mmHg	−6.63 (−11.41 to −1.84)	0.0%	0.027
End-of-treatment office DBP	3	121	MD, mmHg	−0.07 (−9.54 to 9.39)	71.0%	0.976
Change in office SBP	4	360	MD, mmHg	−7.78 (−16.47 to 0.91)	88.8%	0.065
Change in office DBP	4	360	MD, mmHg	−3.97 (−7.26 to −0.68)	69.7%	0.031
24-h ambulatory SBP, final	2	168	MD, mmHg	−1.22 (−57.07 to 54.64)	75.6%	0.828
24-h ambulatory DBP, final	2	168	MD, mmHg	−1.19 (−19.35 to 16.97)	6.7%	0.557
24-h ambulatory SBP, change	4	544	MD, mmHg	−3.60 (−10.35 to 3.16)	70.8%	0.189
24-h ambulatory DBP, change	4	544	MD, mmHg	−3.05 (−5.57 to −0.53)	0.0%	0.031
Blood pressure control or response	Not pooled	Not pooled	RR or narrative	Narrative only	–	Not applicable
Heart rate variability	Not pooled	Not pooled	SMD or narrative	Narrative only	–	Not applicable
Health-related quality of life	Not pooled	Not pooled	SMD or narrative	Narrative only	–	Not applicable
Sleep or anxiety outcomes	Not pooled	Not pooled	SMD or narrative	Narrative only	–	Not applicable

CI, confidence interval; DBP, diastolic blood pressure; HRV, heart rate variability; MD, mean difference; RR, risk ratio; SBP, systolic blood pressure; SMD, standardized mean difference. Negative MD values favor acupuncture-based interventions. Very wide confidence intervals in two-study ambulatory final-value analyses reflected sparse data and Hartung–Knapp adjustment.

### 3.6 Subgroup analyses and extended Acupoint stimulation analysis

**Figure 4:**
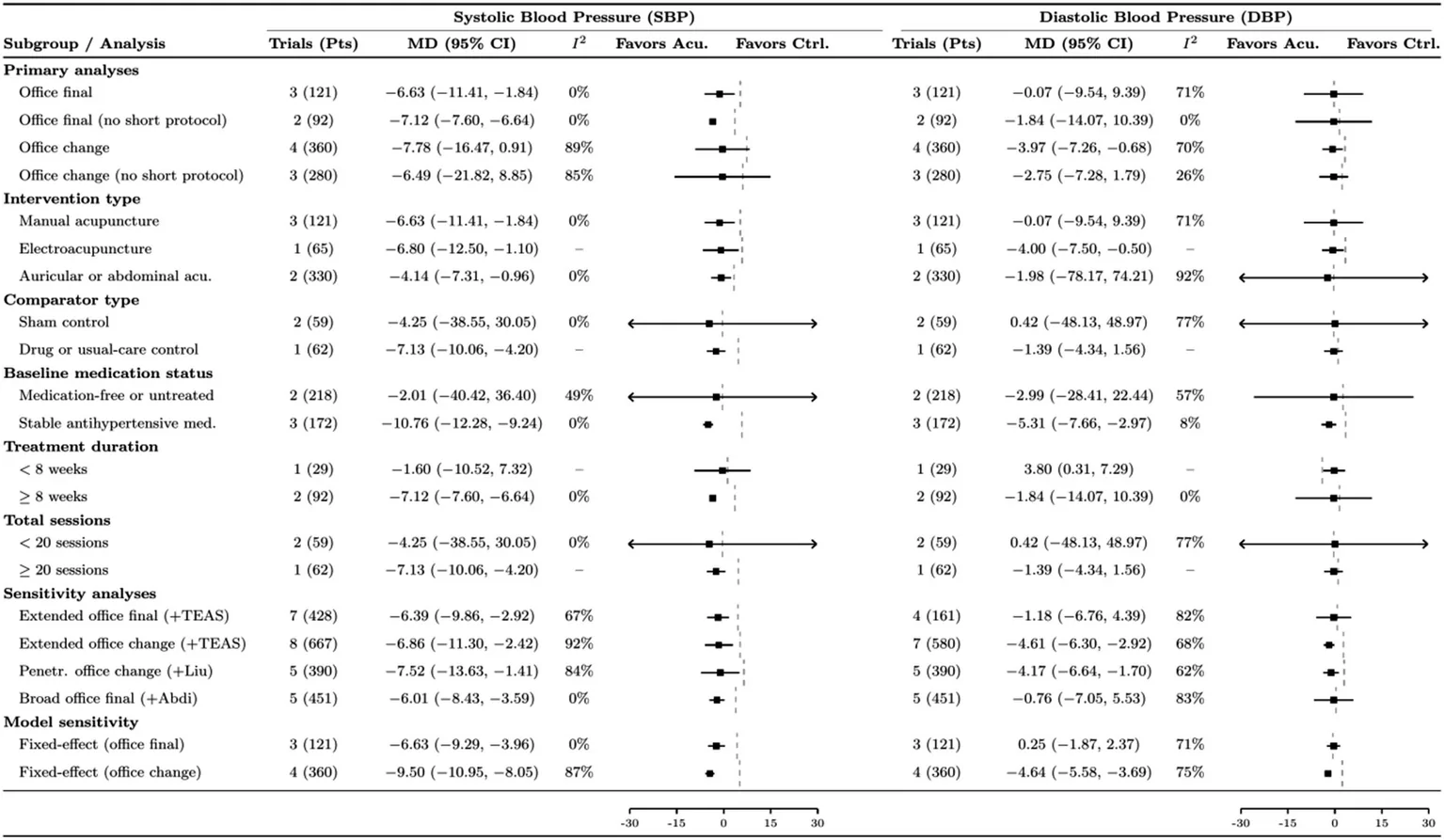
Summary forest plots of subgroup, extended-intervention, and sensitivity analyses for blood pressure outcomes. CI, confidence interval; DBP, diastolic blood pressure; MD, mean difference; SBP, systolic blood pressure; TEAS, transcutaneous electrical acupoint stimulation. Negative MD values favor the intervention group. Single-study groups are shown descriptively without heterogeneity metrics. The graphical forest-plot limits are set at 30 mmHg; confidence intervals exceeding these limits are indicated by truncated arrows.

Subgroup, extended-intervention, and sensitivity analyses are summarized in Figure 4. For the primary office final-value analysis, exclusion of short-protocol studies produced a similar SBP estimate (MD −7.12 mmHg, 95% CI: −7.60 to −6.64), while the DBP estimate remained imprecise (MD −1.84 mmHg, 95% CI: −14.07 to 10.39). In the office change-score analysis, the direction of effect remained favorable after exclusion of short-term studies, although confidence intervals widened for both SBP and DBP. Subgroup analyses by intervention type showed that manual acupuncture was consistent with the primary office final-value analysis. Electroacupuncture was represented by one study and showed reductions in 24-h average SBP and DBP. Auricular or abdominal acupuncture was retained only as broad sensitivity evidence; the SBP estimate favored intervention, whereas the DBP estimate was highly imprecise. Subgroup analyses by comparator type showed wider confidence intervals among sham-controlled studies, while the drug or usual-care control subgroup was represented by a single study and was interpreted descriptively. Analyses by baseline medication status suggested larger office blood pressure reductions among participants receiving stable antihypertensive medication than among medication-free or untreated participants. Treatment-exposure analyses showed directionally favorable SBP estimates for studies lasting 8 weeks or longer and for studies with 20 or more sessions; however, these comparisons were based on a small number of studies and were not interpreted as definitive evidence of a dose–response relationship. Extended analyses including TEAS studies produced results broadly consistent with the primary findings. For office final values, the extended analysis showed lower SBP (MD −6.39 mmHg, 95% CI: −9.86 to −2.92), while DBP remained imprecise (MD −1.18 mmHg, 95% CI: −6.76 to 4.39). For office change scores, the extended analysis showed reductions in both SBP (MD −6.86 mmHg, 95% CI: −11.30 to −2.42) and DBP (MD −4.61 mmHg, 95% CI: −6.30 to −2.92), although heterogeneity was substantial. Sensitivity analyses supported the direction of the main findings. Inclusion of the elevated-BP Liu et al. study in the penetrating office change analysis yielded favorable estimates for both SBP and DBP. Broad office final-value sensitivity analysis including Abdi et al. also favored intervention for SBP but not for DBP. Fixed-effect model analyses produced stronger and more precise pooled estimates; however, because clinical and methodological heterogeneity was expected across intervention protocols, comparator types, and study populations, the random-effects model with Hartung–Knapp adjustment was retained as the primary analytical approach.

### 3.7 Safety, small-study effects, and certainty of evidence

Safety and tolerability outcomes are summarized in Table 5. Adverse-event monitoring was reported in most studies, but the level of detail varied substantially. No confirmed acupuncture- or stimulation-related serious adverse event was clearly reported. Reported events were generally mild, local, or transient, including needling pain, discomfort, spot bleeding, hematoma, fear of needle sensation, itchiness, or numbness. In Macklin et al. (32), serious adverse events occurred during follow-up but were not clearly acupuncture-specific. In Flachskampf et al. (33), two participants discontinued active acupuncture because needling was too painful. Overall, the safety profile did not suggest a clear excess risk from acupuncture-based interventions, but incomplete adverse-event ascertainment limited the strength of this conclusion. Small-study effects were not formally assessed using funnel plots or regression-based asymmetry tests because no pooled outcome included at least 10 independent comparisons. Publication bias was therefore considered qualitatively within the GRADE assessment rather than statistically tested. The certainty of evidence is summarized in Table 6. Evidence certainty was rated as low for end-of-treatment office SBP and change in office DBP, and very low for end-of-treatment office DBP, change in office SBP, ambulatory blood pressure outcomes, narrative cardiovascular risk-related outcomes, and safety. The main reasons for downgrading were risk of bias, inconsistency, imprecision, sparse data, heterogeneous outcome definitions, and incomplete safety reporting. These ratings indicate that the observed blood pressure effects, particularly for office SBP and ambulatory DBP change, should be interpreted as suggestive rather than definitive.

**Table 5:** Safety and tolerability outcomes across included studies.

Study	Acupuncture- or stimulation-related events	Serious events and discontinuation
Macklin et al. (32)	Study-related adverse events were monitored; no clear excess acupuncture-specific signal was identified	Three study-related serious adverse events occurred during follow-up but were not clearly acupuncture-specific; discontinuation was not clearly attributable to acupuncture in the pooled summary
Flachskampf et al. (33)	Needling pain or discomfort was reported by some participants	No serious adverse events were reported; two active-acupuncture participants discontinued because acupuncture was too painful
Yin et al. (34)	Spot bleeding was reported in real-acupuncture sessions	No serious adverse events were reported; dropouts occurred before or after treatment and were not primarily safety-attributed
Kim et al. (35)	No clear count extractable	Serious adverse events were not clearly reported; five randomized participants were not analyzed
Liu et al. (36)	No adverse events reported	No serious adverse events or adverse-event-related discontinuations were reported
Li et al. (37)	No complications or adverse effects reported	No serious adverse events were reported; treatment discontinuation was not clearly reported
Abdi et al. (38)	No severe or systemic adverse effects reported	No serious adverse events were reported; withdrawals occurred overall, but reasons were not primarily acupuncture-specific
Zheng et al. (39)	Active acupuncture: five events; sham acupuncture: four events	No serious adverse events or major safety-related discontinuations were reported
Huang et al. (40, 41)	No obvious adverse effect; three participants reported fear of needle sensation	No serious adverse events were reported; eight participants withdrew overall, but no withdrawal was clearly attributed to acupuncture-related harm
Maheshkumar et al. (42)	No specific adverse events reported	Serious adverse events and treatment discontinuation were not clearly reported
Zhang et al. (43)	No actual event count clearly reported	Serious adverse events and treatment discontinuation were not clearly reported
Tu et al. (44)	No TEAS-related adverse events occurred	No serious adverse events or TEAS-related discontinuations were reported
Ma et al. (45)	No adverse events reported in the trial summary	No serious adverse events or adverse-event-related discontinuations were reported
Bao et al. (46)	Mild stimulation-related events were reported, including transient itchiness or numbness	No serious adverse events were reported; dropout rate was reported, and treatment-related events were mild
Emara et al. (47)	No obvious adverse effects reported	No serious adverse events were reported; treatment discontinuation was not clearly reported

TEAS, transcutaneous electrical acupoint stimulation. Study-related serious adverse events were distinguished from confirmed acupuncture-related serious adverse events. The absence of reported adverse events was not interpreted as evidence of absence of harm when adverse-event monitoring was not explicitly described. Overall, serious acupuncture- or stimulation-related adverse events were not clearly reported, and most described events were mild, local, or transient.

**Table 6:** GRADE summary of findings.

Outcome	Studies	Participants	Effect estimate	Certainty
End-of-treatment office SBP	3	121	MD −6.63 (−11.41 to −1.84) mmHg	Low
End-of-treatment office DBP	3	121	MD −0.07 (−9.54 to 9.39) mmHg	Very low
Change in office SBP	4	360	MD −7.78 (−16.47 to 0.91) mmHg	Very low
Change in office DBP	4	360	MD −3.97 (−7.26 to −0.68) mmHg	Low
24-h ambulatory SBP change	4	544	MD −3.60 (−10.35 to 3.16) mmHg	Very low
24-h ambulatory DBP change	4	544	MD −3.05 (−5.57 to −0.53) mmHg	Very low
Blood pressure control or response	Not pooled	Not pooled	Narrative only	Very low
Heart rate variability	Not pooled	Not pooled	Narrative only	Very low
Health-related quality of life	Not pooled	Not pooled	Narrative only	Very low
Safety and tolerability	15	Not pooled	Narrative only	Very low

Randomized controlled evidence started as high-certainty evidence and was downgraded for risk of bias, inconsistency, indirectness, imprecision, and publication bias. DBP, diastolic blood pressure; HRV, heart rate variability; MD, mean difference; SBP, systolic blood pressure.

## 4 Discussion

### 4.1 Principal findings and comparison with previous evidence

This systematic review and meta-analysis of randomized evidence found that acupuncture-based interventions were associated with a favorable reduction in office SBP, whereas effects on office DBP were less consistent. The primary end-of-treatment office analysis showed a significant reduction in SBP, while office DBP showed substantial uncertainty. Change-score analyses suggested favorable effects for DBP and directionally favorable effects for SBP. Ambulatory BP evidence was more limited: 24-h ambulatory DBP change favored acupuncture-based interventions, but ambulatory SBP change remained imprecise. These findings are clinically relevant because even modest reductions in SBP can translate into meaningful reductions in cardiovascular events at the population level (3–6). Our results are broadly consistent with earlier reviews suggesting that acupuncture may have BP-lowering potential, particularly when used as an adjunctive intervention; however, our interpretation is more conservative because we restricted the evidence base to randomized reports and separated penetrating acupuncture, electroacupuncture, TEAS, office BP, ambulatory BP, final values, and change scores (10–12, 16). Recent reviews have reported favorable results for primary hypertension and 24-h ambulatory BP, but many often pooled clinically diverse acupuncture-related interventions (13–15).

### 4.2 Potential pathophysiological mechanisms and differential blood pressure effects

Hypertension is sustained by interacting neural, vascular, renal, and neurohumoral mechanisms, including sympathetic overactivity, impaired baroreflex regulation, activation of the renin–angiotensin–aldosterone system, endothelial dysfunction, increased peripheral vascular resistance, and progressive arterial stiffening. Acupuncture has been proposed to influence several of these pathways through somatic afferent stimulation and subsequent modulation of hypothalamic and brainstem cardiovascular networks. Experimental evidence suggests that acupuncture or electroacupuncture may engage the arcuate nucleus, ventrolateral periaqueductal gray, medullary raphe regions, and rostral ventrolateral medulla, with endogenous opioid and gamma-aminobutyric acid signaling contributing to attenuation of sympathoexcitatory responses. These effects could reduce sympathetic outflow, modify autonomic balance, and influence peripheral vascular tone and stress-related cardiovascular reactivity (7, 8). However, much of this mechanistic evidence derives from experimental models or small physiological studies, and the trials included in this review were not designed to establish whether these pathways mediated the observed blood pressure changes.

The differing findings for SBP and DBP may reflect both their distinct hemodynamic determinants and the structure of the available evidence. SBP is influenced strongly by stroke volume, large-artery stiffness, and arterial wave reflection, whereas DBP is more closely related to peripheral vascular resistance, heart rate, and arterial recoil during diastole (4, 5). A reduction in sympathetic vasoconstriction or peripheral resistance could therefore be expressed more consistently as a change in DBP, while changes in cardiac output, stress reactivity, or pulsatile arterial load may be more apparent in office SBP. Nevertheless, the observed pattern should not be interpreted as evidence that acupuncture selectively affects one blood pressure component. The significant estimates for end-of-treatment office SBP, office DBP change, and ambulatory DBP change were derived from partly different study sets and outcome formats. Differences in baseline blood pressure, age-related arterial stiffness, medication status, acupuncture protocol, comparator design, and measurement context may also have contributed. Moreover, several analyses contained few studies, wide confidence intervals, or substantial heterogeneity. The apparent differences between SBP and DBP findings therefore most likely reflect a combination of potentially distinct physiological responses and limitations of the current evidence rather than a confirmed blood-pressure-specific mechanism.

### 4.3 Clinical interpretation and cardiovascular risk implications

The findings should be interpreted in the context of contemporary hypertension care, where non-pharmacological strategies are recommended as part of comprehensive BP management but should not replace evidence-based antihypertensive therapy when drug treatment is indicated (4, 5, 9). The observed office SBP reduction suggests that acupuncture-based interventions may be considered as supportive, patient-preference-sensitive approaches, especially for patients interested in complementary non-pharmacological care. Nevertheless, the evidence does not support acupuncture as a stand-alone cardiovascular risk reduction strategy. Its role is more appropriately framed as an adjunct to lifestyle modification, usual care, and pharmacological management. Ambulatory BP outcomes are particularly important because out-of-office BP is closely linked to cardiovascular risk assessment and treatment evaluation (5, 28). The limited and heterogeneous ambulatory evidence in this review indicates that future studies should prioritize 24-h ABPM, nocturnal BP, BP variability, and circadian BP patterns. Mechanistically, acupuncture may influence BP through autonomic regulation, modulation of sympathetic outflow, neurohumoral pathways, and central autonomic integration, but these mechanisms remain supportive rather than definitive clinical evidence (7, 8). TEAS may share some acupoint-based neuromodulatory features, but it is not equivalent to needle acupuncture and should be evaluated as a related but distinct intervention.

### 4.4 Clinical and methodological heterogeneity

Clinical heterogeneity across the included studies extended beyond the distinction between penetrating acupuncture and non-penetrating acupoint stimulation. Manual acupuncture depends on needle insertion, manipulation, and the resulting sensory stimulation, whereas electroacupuncture superimposes a controlled electrical current and may provide a more standardized stimulation intensity. TEAS delivers electrical stimulation without needle penetration and was therefore treated as a related but distinct intervention in the extended analyses. Treatment exposure also varied markedly, ranging from a single short-term session to 12-week treatment courses, from once weekly to five sessions per week, and from 1 to 48 planned sessions. Greater cumulative exposure could plausibly produce more sustained autonomic or vascular effects, whereas single-session and short-protocol studies primarily capture immediate physiological responses. Although subgroup estimates appeared more favorable in some studies with treatment durations of 8 weeks or longer or with 20 or more sessions, these comparisons involved few studies and were confounded by differences in modality, population, medication status, and comparator design. They therefore should not be interpreted as establishing a dose–response relationship.

Acupoint selection introduced an additional source of heterogeneity. The included trials used fixed or individualized prescriptions involving ST36, PC6, LR3, KI3, LI4, LI11, and abdominal or auricular acupoint regions. These choices reflect different therapeutic rationales and may engage overlapping but not identical somatosensory and autonomic pathways. Moreover, acupoint selection was delivered as part of a broader treatment package that also included needle depth, manipulation, electrical stimulation, retention time, treatment frequency, and co-interventions. Consequently, the observed effects cannot be attributed to any individual acupoint, and protocols centered on ST36 or PC6 are not necessarily directly comparable with short-protocol LR3–KI3 stimulation or multichannel electroacupuncture. The pooled estimates should therefore be interpreted as average effects across heterogeneous acupuncture regimens rather than as evidence for a single standardized intervention (10–12, 19).

Comparator design further affected the meaning and comparability of the treatment effects. The included studies used sham acupuncture, non-acupoint needling, control-acupoint stimulation, sham electrical stimulation, waiting-list or no-treatment controls, usual care, and antihypertensive treatment alone. Blinding practitioners to the intervention is generally not feasible, and the success of participant blinding was not consistently assessed. In addition, penetrating or non-penetrating sham procedures may not be physiologically inert because they can generate somatosensory input, treatment expectations, attention, and other contextual effects. Such effects may reduce the observed difference between genuine and sham acupuncture. Conversely, waiting-list, no-treatment, or usual-care controls do not fully match practitioner contact, treatment time, or expectations and may produce larger apparent between-group differences. Accordingly, sham-controlled comparisons more closely estimate the effect of the specific acupuncture protocol beyond a credible procedural control, whereas usual-care or no-treatment comparisons estimate the overall effect of adding acupuncture-related care. Pooling these different contrasts may contribute to heterogeneity and limits the extent to which a single pooled estimate can be generalized across clinical settings. These considerations are consistent with the observed risk-of-bias concerns and the low or very low GRADE certainty ratings and support a cautious interpretation of the findings.

### 4.5 Safety and long-term outcomes

Across the 15 independent randomized studies, no confirmed acupuncture- or stimulation-related serious adverse event was clearly reported. The treatment-related events that were described were predominantly mild, local, and transient. These included needling pain or discomfort, spot bleeding, hematoma, fear of needle sensation, and transient itchiness or numbness associated with electrical stimulation. Two participants receiving active acupuncture in Flachskampf et al. (33) discontinued treatment because needling was considered too painful. Serious adverse events were reported during follow-up in Macklin et al. (32), but they were not clearly attributable to acupuncture. Several other studies reported no adverse events or no obvious treatment-related complications, although the intensity and completeness of safety monitoring varied across trials.

Overall, these findings suggest a generally favorable short-term safety and tolerability profile when acupuncture is performed by appropriately trained practitioners using suitable patient screening, hygienic needling procedures, and appropriate clinical monitoring (29–31). Nevertheless, the certainty of the safety evidence was rated as very low because adverse-event definitions, surveillance procedures, causal attribution, and reporting of withdrawals were inconsistent. The absence of reported adverse events should not be interpreted as evidence of absence when active safety surveillance was not explicitly described. In addition, most studies recorded adverse events only during treatment or at the end of intervention periods that generally lasted 4 to 12 weeks, and few provided extended post-treatment surveillance. The available evidence therefore cannot establish long-term safety, the durability of blood pressure reduction, or effects on cardiovascular morbidity and mortality. Future trials should incorporate standardized adverse-event definitions, prospective safety monitoring, explicit assessment of event causality and severity, and longer follow-up.

### 4.6 Strengths, limitations, and future research

A major strength of this review is its deliberate separation of evidence streams. We distinguished penetrating acupuncture and electroacupuncture from non-penetrating TEAS, analyzed office and ambulatory BP separately, separated final-value and change-score outcomes, and used subgroup, extended-intervention, and sensitivity analyses to examine the robustness of the findings. This structured approach made the principal sources of clinical and methodological heterogeneity more transparent, although it could not eliminate the underlying differences among intervention protocols and comparator designs. We also summarized safety and certainty of evidence explicitly rather than relying only on pooled efficacy estimates. This design improves interpretability for cardiovascular readers and aligns with recent calls to evaluate non-pharmacological hypertension interventions using clinically meaningful BP outcomes and transparent evidence grading (5, 9, 13).

Several limitations should be acknowledged. The number of trials contributing to each pooled outcome was small, and several analyses were based on few comparisons. Risk of bias was common, particularly because participant blinding and intervention concealment are difficult in acupuncture trials. Some outcomes were limited by imprecision and high heterogeneity. In addition, the available trials were generally not designed or powered to evaluate sustained blood pressure control or cardiovascular events. Future trials should use adequately powered multicenter designs, credible sham or attention-control comparators, standardized office and ambulatory BP measurement, longer follow-up, and prespecified core outcomes.

## 5 Conclusion

This systematic review and meta-analysis suggests that acupuncture-based interventions may provide modest support for blood pressure control in adults with hypertension, particularly for end-of-treatment office systolic blood pressure and diastolic blood pressure change outcomes. Evidence from ambulatory blood pressure outcomes was more limited, although 24-h ambulatory diastolic blood pressure change also favored acupuncture-based interventions. No confirmed acupuncture- or stimulation-related serious adverse event was clearly identified, but safety reporting was inconsistent across studies. Overall, acupuncture-based interventions may be considered as supportive non-pharmacological approaches rather than substitutes for established hypertension treatment. Future randomized trials should use rigorous sham or attention-control designs, standardized office and ambulatory blood pressure measurement, longer follow-up, and systematic safety assessment to clarify the clinical role of acupuncture-based interventions in hypertension management.

## Data Availability

The original contributions presented in the study are included in the article/supplementary material, further inquiries can be directed to the corresponding author.
